# Meaning in life, meaning-making and posttraumatic growth in cancer patients: Systematic review and meta-analysis

**DOI:** 10.3389/fpsyg.2022.995981

**Published:** 2022-12-09

**Authors:** Margarida Almeida, Catarina Ramos, Laura Maciel, Miguel Basto-Pereira, Isabel Leal

**Affiliations:** ^1^Ispa – Instituto Universitário, Lisbon, Portugal; ^2^LabPSI – Laboratório de Psicologia Egas Moniz, Centro de Investigação Interdisciplinar Egas Moniz, Instituto Universitário Egas Moniz, Caparica, Portugal; ^3^WJCR – William James Center for Research, Ispa – Instituto Universitário, Lisbon, Portugal

**Keywords:** meta-analysis, meaning, posttraumatic growth, psycho-oncology, cancer, systematic review, cancer survivorship

## Abstract

**Introduction:**

The purpose of this systematic review and meta-analysis is to assess the association between meaning in life (MiL), meaning-making and posttraumatic growth (PTG) in the context of cancer.

**Methods:**

A systematic search was conducted in eighteen electronic databases. The screening and selection process followed the PRISMA guidelines. For the purpose of the meta-analysis, the correlation coefficients between meaning in life and posttraumatic growth were extracted from the included studies. The effect size (*r*) was calculated using the restricted maximum-likelihood estimator, a random-effects model. Heterogeneity was assessed through the *Q* statistic, *I^2^* index and forest plot, while publication bias was analyzed with the use of the funnel plot and Egger’s test.

**Results:**

889 records were considered according to the inclusion criteria. A total of nine articles, published between 2006 and 2021, were included in the systematic review. More than half were published in the last five years. The sample was mostly diagnosed with breast cancer. The meta-analysis included five articles (*N* = 844) and the results indicate a significant moderate correlation between meaning in life and posttraumatic growth (*r* = 0.43, 95% IC [0.36, 0.50]).

**Discussion:**

In conclusion, there is a clear association between meaning in life and posttraumatic growth in cancer patients. Future research should explore this relationship further, in order to better assist and guide meaning centered interventions that can potentiate a positive adjustment and possibly growth from the cancer experience.

## Introduction

Cancer is one of the leading causes of death in most countries (Bray et al., 2021). The World Health Organization (WHO) estimated that there were approximately 19.3 million new cases and nearly 10 million deaths of cancer in 2020 (World Health Organization, 2021). Cancer is a long process that goes through different stages, from the diagnosis and treatment to survivorship, with a variety of implications in the lives of cancer patients (Holland, 2002, 2003). For that reason, these patients are particularly at risk of experiencing psychological distress related to feelings of anxiety, depression, fear or guilt (Costa et al., 2016). There is therefore a higher risk for cancer patients to develop mental disorders (Singer et al., 2010), which impact their quality of life and can increase cancer-specific mortality by 53% (Kuhnt et al., 2016; Zhu et al., 2017). Due to its prevalence, the research on the psychological outcomes of cancer has predominantly focused on anxiety and depression (e.g., Singer et al., 2010, 2013; Kuhnt et al., 2016; Zhu et al., 2017; Michel et al., 2019). However, over the years the focus has started to shift to coping mechanisms and positive emotional outcomes (Johansson et al., 2011), such as psychological adjustment, benefit-finding and posttraumatic-growth (Costa et al., 2016; Singer, 2018).

### Posttraumatic growth

Posttraumatic growth (PTG) is the psychological growth that arises from the struggle with stressful life event (Tedeschi and Calhoun, 1995). In the book *Trauma and Transformation*, Tedeschi and Calhoun (1995) describe how psychological growth derives from a change in perspective that results “in a more profound understanding of the self and world” (p. 87). The change is considered to be transformative, seeing has it occurs at a cognitive and emotional level, which in turn leads to changes in behavior (Tedeschi et al., 2018).

PTG is a long-term change triggered by a traumatic event, considered as a highly stressful life-changing experience (Tedeschi et al., 2018), such as an illness like cancer (Tedeschi and Calhoun, 2004). The struggle that comes from trying to cope and overcome these kind of experiences, is what initiates the process of PTG (Tedeschi and Calhoun, 1995). The Model of PTG describes this process where after a highly stressful and possibly traumatic event there can be a disruption of a person’s core beliefs, causing emotional distress which leads to intrusive rumination. Once these intrusive thoughts are transformed into constructive and deliberate thoughts, an acceptance of the changed world will be possible as the meaning attributed to the event will facilitate its integration in a person’s life narrative. The combination of challenged core beliefs, rumination and distress is what promotes the experience of PTG (Tedeschi et al., 2018). There are five possible domains where change may occur due to this process: personal strength, relating to others, new possibilities, appreciation of life and spiritual and existential change (Tedeschi and Calhoun, 2004).

In cancer patients, PTG has been consistently related to a better quality of life (Liu et al., 2020; Kim and Son, 2021) and to lower levels of depression and anxiety (Thakur et al., 2022). Considering that the changes of PTG can be seen as some of the best outcomes of the cancer experience due to their impact on the lives of cancer survivors, empirical research has thoroughly examined its correlates and predictors (Shand et al., 2015; Casellas-Grau et al., 2017; Turner et al., 2018). According to the literature, one of the correlates and predictors of PTG seems to be in fact meaning in life (Casellas-Grau et al., 2017). Additionally, as stated by Tedeschi et al. (2017), existential concerns, such as spirituality and questions regarding life’s meaning, are a part of the process of change experienced after a highly stressful life-changing experience.

### Meaning in life

Meaning is an integrant part of the human existence (Steger, 2012b). People need a sense of meaning in their lives (Frankl, 1946), in order to understand their existence and to feel that it is significant and purposeful (Steger and Kashdan, 2007). Since Frankl inaugurated the psychological study on the meaning in life (Baumeister and Vohs, 2002), a growing body of literature has explored the importance and role of meaning in people’s life. However, despite the increasing interest, the concept of meaning in life (MiL) is still reason for discussion and debate (Heintzelman and King, 2014). Research on the MiL originated in a variety of definitions and theoretical models (Martela and Steger, 2016), which in turn has resulted in the development of several instruments.

MiL is a multidimensional construct composed of three components: purpose, significance and comprehension or coherence (Steger, 2018). Purpose, the motivational component, refers to a set of core goals and aims that give a sense of direction to life (George and Park, 2013; Martela and Steger, 2016; Steger, 2018). Significance is the evaluative and affective component of meaning. It involves the value, worth and importance a person attributes to their life’s, giving it meaning (Martela and Steger, 2016). In other words, it is the feeling of a significant and relevant existence, that is ultimately considered to be *worth living* (George and Park, 2016). The third and cognitive component is termed as comprehension or coherence (Martela and Steger, 2016). It refers to the ability to make sense of life and the world through “a web of connections, understandings, and interpretations that help us comprehend our experience” (Steger, 2012b, p. 165). MiL is therefore a set of subjective judgments people make of their lives. It involves having a sense of significance and feeling that their own lives matter, perceiving life as comprehensible and coherent, and having a sense of purpose (King et al., 2006; Steger, 2012a, 2018).

Meaning is considered to be one of the key components for a good mental health (Fusar-Poli et al., 2020) and psychological well-being (Fischer et al., 2021). In cancer patients, the diagnosis and treatment can evoke concerns regarding MiL, as the idea of one’s mortality comes into mind (Carreno and Eisenbeck, 2022). When people perceived their life’s as meaningful, they experience less distress (Winger et al., 2016), facilitating their adjustment to the illness. This might be explained by the fact that MiL influences a cancer patients’ perception of their illness (Krok and Telka, 2017), consequently impacting the use of coping strategies (Miao and Gan, 2020), which in turn affects the psychological outcomes and emotional experience crucial for a positive or negative adjustment. Empirical research has shown that higher levels of MiL correlate to lower levels of depression and anxiety (Vehling et al., 2011; Elekes, 2017; Testoni et al., 2018; Gravier et al., 2020) and distress (Jaarsma et al., 2007; Winger et al., 2016).

Receiving a diagnosis of cancer frequently triggers a process of search for meaning (Park et al., 2008). This process is described by the meaning-making model, where it is shown the role of meaning in coping and adjusting to stressful events (Park, 2010). According to this model there are two types of meaning – global meaning and situational meaning – and when faced with an adversity, such as the diagnosis of cancer, there may occur a meaning discrepancy caused by the difficulty in incorporating the illness (situational meaning) into one’s overall MiL (global meaning; Park and Folkman, 1997). The discrepancy provokes distress, which will trigger meaning-making (MM) efforts to restore or rebuilt the meaning systems (Park, 2010).

Some authors do not properly distinct the concept of MM from PTG, since both share common points and similar paths in the trajectory of cancer (Casellas-Grau et al., 2017). However, MM can be considered as the search for meaning in face with adversity (Steger, 2018), while PTG is the positive personal transformation that derives from the struggle with a highly stressful event.

Considering the role that both MiL and PTG have on the adjustment and outcomes of the cancer experience, it might be relevant to understand how they interact in these patients in particular. In fact, MiL has been one of the variables that has shown to be positively correlated and related to PTG in different contexts and samples, including cancer patients. However, despite the variety of systematic reviews that assess both of these variables independently, to our knowledge there is no systematic review or meta-analysis that summarizes and analyzes the relationship between MiL and MM to PTG in the cancer population. A recent systematic review suggested that MiL and PTG could be associated in cancer patients (Casellas-Grau et al., 2017). However, there was no clear distinction in the differences between these two concepts, which lead for instance to the analysis of articles that considered both concepts to be synonyms. Only by making a clear distinction between MiL an PTG, will it be possible to truly understand how these concepts relate, which can benefit and guide the development of interventions for cancer patients.

Systematic reviews and meta-analysis inform the readers of relevant studies and their results, while also providing the necessary data to assist and guide policies and clinical practice. The goal of a systematic review with a meta-analysis is to combine and synthesize the empirical research on a particular topic in order to answer a specific question (Littell et al., 2008). “How are meaning in life and meaning-making related to PTG in cancer patients?” is the main question that this systematic review aims to answer. In addition to revealing the existent research on the relationship between MiL, MM and PTG in cancer, the objectives of this paper are to: a) analyze the similarities and differences between studies and to b) examine how MiL, MM and PTG impact the lives of cancer patients. The objective specific to the meta-analysis is to assess how strong is the association between MiL and PTG in cancer patients.

## Materials and methods

The systematic review and meta-analysis protocol was registered in the International Prospective Register of Systematic Reviews (PROSPERO) and published on December 16, 2021 with the registration number CRD42021287048.

### Eligibility criteria

The inclusion and exclusion criteria were delineated before the development of the search strategy, according to the objectives of this paper. The type of studies that were considered for inclusion were quantitative, comparative, correlational, cross-sectional, longitudinal studies or randomized controlled trials. Mixed-method studies were included only if they performed a quantitative analysis of the main variables. In the meta-analysis only were included cross-sectional studies. All the articles had to be published in a peer-reviewed journal and contain at least an English or Portuguese abstract. If there wasn’t an English version of the article, the author(s) were contacted to request additional information.

Empirical studies that examined the primary outcomes of this review, PTG and MiL and/or MM in adult cancer patients as either a primary or secondary outcome were considered for inclusion. *Posttraumatic Growth* are the positive psychological changes experienced as a result of a traumatic or challenging experience (Tedeschi and Calhoun, 1995, 2004). To measure PTG the studies should apply a validated instrument, such as the Posttraumatic Growth Inventory (PTGI), Perceived Benefits Scale (PBS), or the Benefit Finding Scale (BFS). *Meaning in Life* is a feeling of significance and importance of one’s life. It involves perceiving life as comprehensible and with purpose (Steger, 2018). There are several validated instruments that assess MiL (Brandstätter et al., 2012), the most recent studies apply the Meaning in Life Questionnaire (MILQ). *Meaning-Making* is a model, developed by Park and Folkman (1997), that describes the role that meaning has in a person’s adjustment to stressful life events. So far, no instrument was developed to assess specifically the process of MM. Regarding the illness, there were no restrictions on the type of cancer, the cancer stage, time since diagnosis or the cancer treatment. In order to be included in the meta-analysis, studies had to present statistical data allowing the calculation of the effect size. If the published study did not present the necessary information, the author(s) were contacted to request the data.

The exclusion criteria included reviews, meta-analysis, theoretical articles, study protocols, books and chapters of books. Qualitative articles, validation of instruments and interventions, or medical articles were excluded. Studies were also excluded if they did not use an adult sample or if the main variables of this review (PTG and MiL or MM) were not included and assessed with a valid instrument.

### Search strategy

The search for studies was conducted on the 29^th^ of November and the 10^th^ of December of 2021, without restriction on date of publication. The electronic databases (Academic Search Complete, Complementary Index, MEDLINE, APA PsycINFO, ScienceDirect, Psychology and Behavioral Sciences Collection, Supplemental Index, Directory of Open Access Journals, APA PsycArticles, ERIC, Business Source Complete, Criminal Justice Abstracts, Library Information Science & Technology Abst, Scopus, SciELO, RCAAP, PubMed, Bon and Web of Science) were searched for relevant articles published in peer-reviewed journals. A comprehensive search strategy was used, with the combination of the following keywords: cancer or oncological disease or neoplasm or tumor or tumor and PTG or post-traumatic growth or benefit finding or positive life changes or stress-related growth or perceived benefits or existential growth AND meaning* or existential meaning or purpose or meaning-making or meaning making or search* for meaning. The detailed strategy applied in the different databases is available in the Supplementary Table S1. More apprehensive search databases such as Google Scholar were also browsed. In order to find additional studies, the gray literature was searched as the references of the included articles that explored PTG and MiL or MM were also analyzed.

### Study selection

**Figure 1:**
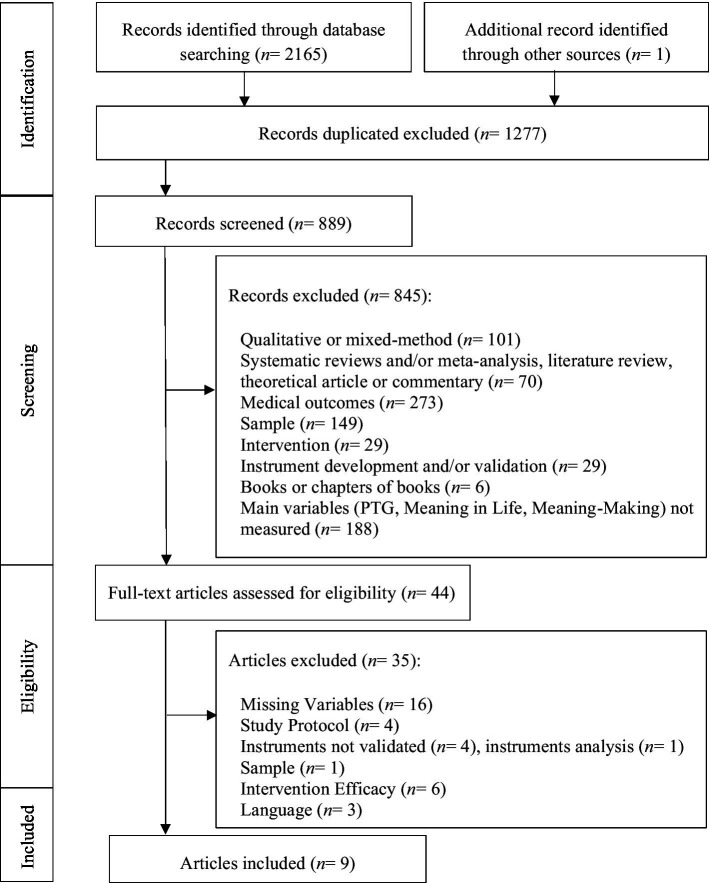
Flow diagram displaying the study selection process based on the PRISMA guidelines.

The articles extracted from the electronic databases were analyzed in accordance with the inclusion criteria. The articles screening and selection process followed the Preferred Reporting Items for Systematic Reviews and Meta-Analyses (PRISMA) guidelines (Page et al., 2021). As recommended, the titles were screened at an initial stage, followed by the analyses of the abstracts, and finally the full text of the articles that met the criteria were obtained and reviewed. A double-screening was performed independently by two reviewers. In the final stage, the reviewers analyzed the full text articles and selected the studies for inclusion. The selected articles were then assessed for their quality by two reviewers independently. The quality assessment followed the criteria for risk of bias assessment of (Shepherd, 2005) for non-intervention studies, later reviewed and adapted by Dancet et al. (2010). Considering that one of the criteria is only applicable for qualitative studies, only six of the seven criteria were applied. Accordingly, each study was attributed a score between zero and six, receiving one point for each of the criteria met and documented (Dancet et al., 2010). In order to ensure the quality of the studies, the minimum score for inclusion was four out of six. Disagreement between reviewers were solved on a consensus-based principle or by the decision of a third independent reviewer. Figure 1 shows the process of study selection.

### Data extraction

Two reviewers independently extracted the necessary data from eligible studies, using a predefined sheet. The study characteristics extracted from the studies were the following: bibliographic information (authors, year of publication and country); sample characteristics (sample size, age, gender, race, relationship status, education); data collection (hospital, data bases and/or social network); study design; cancer characteristics (type of cancer, time since diagnosis); outcomes assessed; measures to assess main outcomes (MiL, MM, and PTG) and the results involving the main outcomes. For the purpose of the meta-analysis, the correlation coefficients and corresponding sample sizes were extracted. Missing characteristics or results were coded as ‘Not Reported’. Disagreement between reviewers were solved on a consensus-based principle.

To ensure the independence of study results, sample characteristics such as country and recruitment source of included studies were closely analyzed. If there were studies that made use of the same sample pool, the study with a larger sample size was preferred. When one study measured the same outcome with different instruments, the most commonly used instrument across studies or the one closest to the concept definition was favored.

### Data analysis

Analysis was conducted using the statistical software JAMOVI (version 2.3, The Jamovi Project, 2022). Considering the objective of this review and that all the variables were continuous, the correlation coefficient (*r*) was used as an effect size metric. Correlations were converted to the Fisher’s Z scale, which was then converted back to correlations for result presentation (Borenstein and Hedges, 2019). Correlation *r* demonstrates the strength and direction of the association between two continuous variables, from −1.0 to 1.0 (Littell et al., 2008). The random-effects model used to estimate the mean effect was the restricted maximum-likelihood estimator (Borenstein et al., 2010). A random-effects model considers that the effect sizes are independent and can vary due to differences in the participants (e.g., age, socioeconomical status, health) or across studies (e.g., study design, treatment conditions) Borenstein et al., 2007; Cheung, 2019). The restricted maximum-likelihood estimator is recommended for meta-analysis with a small number of studies, such as our review (Thompson and Sharp, 1999).

Heterogeneity was assessed through the *Q* statistic and the *I*^2^ index. The *Q* test assesses the presence or absence of heterogeneity between studies, whereas the *I*^2^ index represents the proportion of the total variance due to between-studies variability (Huedo-Medina et al., 2006; Borenstein, 2019). A non-significant *Q* test can indicate that the estimated effect sizes differ due to sampling error alone. However, this statistic has a low sensibility to detect heterogeneity when there is a small number of studies. For that reason, the *I*^2^ index was used to support the *Q* test (Huedo-Medina et al., 2006). An *I^2^* index around 25, 50, and 75% represents a low, medium and high level of heterogeneity, respectively (Higgins, 2003). Additionally, a forest plot was used in order to assess the heterogeneity between studies, as well as the weight of each study to the overall effect.

Publication bias was assessed trough the visual inspection of the funnel plot and confirmed with Egger’s regression test. In the funnel plot when there is publication bias, the distribution of effect sizes will be asymmetrical (e.g., effect sizes clustered in one side of the funnel). However, examination of the funnel plot can be subjective, particularly when there is a small number of studies (Littell et al., 2008). For that reason and to confirm the visual inspection of the funnel plot, Egger’s regression was used. The Egger’s test measures the magnitude and direction of asymmetry, if the test is statistically significant it indicates the presence of publication bias (Vevea et al., 2019).

## Results

### Description of included studies

The literature and additional hand search resulted in 889 potentially relevant articles (after removing duplicates). Following the screening of titles and abstracts, 845 studies were excluded for the following reasons: qualitative or mixed-method, systematic reviews and/or meta-analysis, literature review, theoretical article or commentary, medical outcomes, sample, intervention, instrument development and/or validation, books or chapters of books, and missing variables/outcomes. The remaining 44 eligible articles were reviewed based on a full-text analysis, which resulted in the exclusion of an additional 35 studies (Figure 1; references of excluded articles and reasons for exclusion can be requested from the correspondence author). The data extraction regarding the characteristics of the included studies, including the sociodemographic and clinical characteristics of the sample was summarized in Table 1.

**Table 1:** Details and results of included articles.

Study (Authors, date)	Country	*N* Age *M* (*SD*) Gender (%)	Cancer	Time since diagnosis	Data collection	Study design	PTG scale	Meaning scale	Results
Kallay (2006)	Romania	36 55.66* 64% Female	breast, colorectal, and prostate	4 to 5 months	Cancer Institute	Cross-Sectional	SRGS PTGI	PMP	Personal meaning was positively correlated with SRG (*r* = 0.45, *p* ≤ 0.001) and PTG (*r* = 0.59, *p* ≤ 0.001) There was a moderate positive relationship between negative affectivity and meaning construction (*r* = 0.32, *p* ≤ 0.05) There were significant correlations between most PMP sub-scales and both scales of PTG. One of the highest, significant correlations was between religious meaning making subscale (PMPREL) and PTGI (*r* = 0.70, *p* < 0.01), more specifically, the religious dimension of PTG (PTGIEL; *r* = 0.71, *p* < 0.01)
Park et al. (2008)	EUA	250 T1: 45.2* 69% Female	Breast (47%), prostate (12%), colon/rectal (6%), lymph nodes (5%), cervix/uterus (4%), others (24%)	NR	Hospital Data Base	Cross-Sectional and Longitudinal T1 = within 2 years since treatment; T2 = 1 year later	PBS	Brief COPE-PR PPMS	Positive Reframing (MM) and Growth were significantly correlated at Time 1 and 2 (*p* = 0.01). MiL Time 1 was positively correlated with Growth Time 2 (*r* = 0.20, *p* ≤ 0.05). MM Time 1 was not significantly correlated to Growth Time 2 The cross-sectional model revealed that MM coping was indirectly related to restoration of just-world beliefs through its relationships to growth (β = 0.45, *p* ≤ 0.05) and to MiL (β = 0.16, *p* ≤ 0.05). Growth was related to psychological well-being only indirectly through its relationship to MiL (β = 0.29, *p* ≤ 0.05). MiL was related to psychological well-being directly (β = 0.40, *p* ≤ 0.05) The longitudinal model showed that MM Time 1 was directly related to Growth (β = 0.46, *p* ≤ 0.05) and MiL at Time 1 (β = 0.15, *p* ≤ 0.05) and Growth Time 2 (β = −0.26, *p* ≤ 0.05). Growth at Time 1 was related to MiL Time 1 (β = 0.29, *p* ≤ 0.05) and Time 2 (β = −0.19, *p* ≤ 0.05). At Time 2 MM was related to Growth (β = 0.44, *p* ≤ 0.05) and MiL (β = 0.14, *p* ≤ 0.05) MM at Time 1 and 2 explained 33% of the variance of Growth Time 2. Growth Time 1 and MM Time 2 explained 28% of the variance of MiL Time 2
Thuné-Boyle et al., 2011	United Kingdom	155 56 (13.5) 100% Female	breast	Recent diagnosis	Hospital	Cross-Sectional	BFS	Brief COPE-PR	Religiosity/spirituality, strength of faith, private religious/spiritual practices and personal spiritual involvement were significantly and positively related to positive reframing coping (*r* = 0.28, *p* < 0.0005, *r* = 0.28, *p* < 0.001, *r* = 0.30, *p* < 0.0005, *r* = 0.32, *p* < 0.0005 respectively) and benefit finding at 3 months (*r* = 0.37; *r* = 0.41; *r* = 0.32; *r* = 0.38, *p* < 0.0005 respectively) Seeking emotional support, positive reframing and using humor were all significantly related to higher levels of benefit finding at 3 months (*r* = 0.27, *p* < 0.001; *r* = 0.24, *p* < 0.005; *r* = 0.19, *p* < 0.025 respectively)
George and Park (2013)	EUA	T1 = 250 T2 = 167 T2: 46.34 (6.29) 65% Female	Breast (47%), prostate (12%), colon/rectal (6%), lymph nodes (5%), and cervix/ uterus (4%)	*M* = 3.5 years	Hospital Data Base	Longitudinal T1 = within 1 to 3 years of diagnosis; T2 = 1 year later	PBS	PPMS	MiL was positively correlated to PTG (*r* = 0.32, *p* < 0.01, *n* = 152), positive affect (*r* = 0.50, *p* < 0.01), and life satisfaction (*r* = 0.38, *p* < 0.01). MiL was inversely related to posttraumatic depreciation (*r* = −0.31, *p* < 0.01) and negative affect (*r* = −0.21, *p* < 0.01) Time 1 religiousness (*r* = 0.19, *p* ≤ 0.05, *n* = 140) and daily spiritual experiences (*r* = 0.17, *p* ≤ 0.05) were positively correlated with Time 2 MiL. Time 2 MiL was significantly predicted by daily spiritual experiences (β = 0.28, *p* ≤ 0.05, *n* = 158)
Loeffler et al. (2018)	Germany	65 60.5 (11.7) 100% Female	Breast	Close to 1 year	Cancer Center	Longitudinal T1 = 1 year after treatment; T2 = 1 year later	PTGI	MILQ-P	Presence of MiL was related to lower levels of anxiety (*r* = −0.43, *p* < 0.01) and depression (*r* = −0.64, *p* < 0.01), a higher level of satisfaction with life (*r* = 0.44, *p* < 0.01) and better health-related functioning in terms of role functioning (*r* = 0.31, *p* < 0.01), emotional distress (*r* = 0.34, *p* < 0.01) and social functioning (*r* = 0.27, *p* ≤ 0.05) A higher presence of meaning 1 year after therapy predicted lower levels of depression another year later (β = −0.47, *p* < 0.01) Most indicators for well-being were not significantly correlated with PTG
Shand et al. (2018)	Australia	108 56.36 (10.36) 100% Female	Ovarian	*M* = 37.64 months	Social Network	Cross-Sectional	PTGI	Brief COPE-MCC	Higher scores on the three PTSD subscales (avoidance, intrusion, and hyperarousal) and lower scores on three PTG subscales (relating to others, personal strength, and appreciation of life) were associated with higher levels of depressive and anxiety symptoms and avoidant coping Higher PTSD symptoms but lower PTG scores were associated with lower levels of optimism, perceived social support, meaning-centered coping, and quality of life Higher intrusive symptoms and PTG were associated with higher levels of anxiety symptoms, and coping through social support and meaning Lower scores on the PTSD intrusion subscale and higher scores on the spiritual changes PTG subscale were associated with higher levels of depressive symptoms, and lower levels of coping through social support Higher scores on PTG domains relating to others and appreciation of life were associated with higher perceived social support through social support coping
Aflakseir et al. (2018)	Iran	196 52 (12.32) 100% Female	Breast	18 months	Clinic	Cross-Sectional	PTGI	PMI	There was a significant correlation between social support (*r* = 0.37, *p* < 0.001), personal meaning (*r* = 0.33, *p* < 0.001), and PTG Social support (β = 0.19, *p* = 0.05) and meaningfulness (β = 0.26, *p* = 0.03) significantly predicted PTG The model accounted for 34% of the variance in PTG
Moghadam et al. (2021)	Iran	213 52(16) 100% Female	Breast	6 months to 4 years	Hospitals Data Base	Cross-Sectional	PTGI	MLQ-S MLQ-P	PTG was significantly correlated with search for MiL (*r* = −0.18, *p* = 0.01) and presence of MiL (*r* = −0.45, *p* = 0.01). The presence and search for MiL were also correlated (*r* = 0.38, *p* = 0.01) The proposed model, which included positive reappraisal, mental rumination, social support, coping skills, spirituality and religious coping, explained 90% of the PTG variance Presence of MiL (β = 0.25, *p* = 0.001) and search for MiL (β = −0.25, *p* = 0.001) had a significant effect on Positive Re-evaluation. Positive Re-evaluation, in addition to its direct effect on PTG, was a mediator between PTG and core beliefs, positive and negative religious coping, presence and search for MiL, and deliberate rumination Problem-based coping, emotion-based coping, core beliefs, social support, presence of MiL (β = 0.16, *p* = 0.001), and deliberate rumination had a positive and significant effect on PTG. Intrusive rumination, negative religious coping, and search for MiL (β = −0.12, *p* = 0.001) had a negative and significant effect on PTG
Mostarac and Brajković (2022)	Croatia	149 49.18* 70% Female	Breasts (38.2%), lymphatic system (22.1%), mouth, pharynx and larynx (17.4%), others (0.2%)	NR	Social Network	Cross-Sectional	PTGI	MILQ	PTG is positively correlated with the presence of MiL (*r* = 0.44, *p* < 0.01), the search for MiL (*r* = 0.46, *p* < 0.01) and life satisfaction (*r* = 0.46, *p* < 0.01). The search for MiL explained about one-fifth of the variability of occurrence of positive changes The presence of MiL and the search for MiL are positively intercorrelated (*r* = 0.34, *p* < 0.01) and positively correlated with life satisfaction (*r* = 0.37, *p* < 0.01; *r* = 0.65, *p* < 0.01, respectively) 46% of the variance in life satisfaction was explained by the PTG and specific mediator (mediator – MLQ-P: Adjusted *r*^2^ = 46.18, *F*(2, 146) = 62.64, *p* < 0.001 / mediator – MLQ-S: Adjusted *r*^2^ = 45.80, *F*(2, 146) = 19.38, *p* < 0.001) The indirect effect of the PTG on life satisfaction was significant only through the presence of meaning (β = 0.25, *p* ≤ 0.05, standardized SE = 0.07, standardized 95% CI = [0.12, 0.38]). The indirect effect through the presence of meaning in life explained 53.5% of the total effect of the PTG on life satisfaction

*Not reported; PBS, perceived benefits scale; PTG, posttraumatic growth; PTGI, posttraumatic growth inventory; PPMS, perceived personal meaning scale; MiL, meaning in life; MILQ, meaning in life questionnaire; MILQ-P, meaning in life questionnaire – presence; Brief COPE-PR, positive reframing subscale from brief COPE; Brief COPE-MCC, meaning-centered coping subscale from brief COPE; SRG, stress-related growth; SRGS, stress-related growth scale; PMP, personal meaning profile; PMI, personal meaning index; BFS, benefit finding scale.

### Study characteristics

Most of the studies were conducted in Europe (*n* = 4). The remaining studies were conducted in the United States of America (*n* = 2), Asia (Iran, *n* = 2) and Australia (*n* = 1). The studies from the United States shared the same sample. The first study was published in 2006. However, more than half of the studies were published in the last 5 years (*n* = 5).

Regarding the study design, six studies were cross-sectional, one was longitudinal (Loeffler et al., 2018) and two studies applied both a cross-sectional and a longitudinal design (Park et al., 2008; George and Park, 2013). One of the studies with a cross-sectional and longitudinal design (George and Park, 2013), reported only cross-sectional correlations between the MiL and PTG. Due to the fact that only one study had a longitudinal design, the meta-analysis was conducted only with cross-sectional studies.

Data collection was predominantly achieved with the collaboration of hospitals and cancer centers where patients were recently diagnosed and treated for cancer (*n* = 7). The two remaining studies recruited the sample through social networking, by sharing the study in social media and cancer groups.

### Sample characteristics

A total of 1,172 cancer patients were included in the meta-analysis. Approximately 80% of the participants were diagnosed with breast cancer. Regarding sociodemographic characteristic 74% of the participants were female, with a mean age of 53 years old (*SD* = 5.05). Most participants of included studies were in a relationship (71%, *n* = 1,303, *k* = 8) and had a college degree (45%, *n* = 1,148, *k* = 7). Further characteristics are present in Table 1 for consultation.

### Outcome measures

PTG was more commonly assessed through the Posttraumatic Growth Inventory (PTGI, *n* = 6). Other measures used for PTG were the Perceived Benefits Scale (*n* = 2), the Benefit Finding Scale (*n* = 1), and the Stress Related Growth Scale (*n* = 1).

Regarding MiL, there are a number of instruments that measure the concept, the most recent articles applied the Meaning in Life Questionnaire (*n* = 3). The authors also applied the Perceived Personal Meaning Scale (*n* = 2), the Personal Meaning Profile (*n* = 1) and the Personal Meaning Index (*n* = 1) to measure MiL. Regarding meaning-making, there has not been developed and validated an instrument to measure the process. The authors chose therefore to apply the positive reframing coping subscale of Brief COPE.

Concerning the correlation between variables, two studies did not establish a direct correlation between MiL and PTG (Loeffler et al., 2018; Shand et al., 2018). On the other hand, some studies, through regression analysis, showed a direct (*n* = 3) or indirect (*n* = 1) relationship between MiL and PTG. Other outcomes were also considered for their possible connection to MiL and PTG, such as life satisfaction (*n* = 3), anxiety and depression (*n* = 2). Additionally, researchers showed a tendency to assess the association between PTG and social support (*n* = 3), and between MiL and religion or spirituality (*n* = 4).

### Literature overview

Table 1 shows the main results involving MiL, MM, and PTG obtained by the 9 studies included. The majority of the studies, except for two, assess the correlation between either MiL or MM to PTG. Four of them go beyond by assessing the direct or indirect relationship between these variables.

All studies found a positive significant correlation between MiL and PTG. Two studies found a direct significant effect of MiL in PTG (Aflakseir et al., 2018; Moghadam et al., 2021). Moghadam et al. (2021) shows that the relationship between MiL and PTG is positive for the presence of meaning in life (β = 0.16, *p* = 0.001), but negative for the search for meaning (β = −0.12, *p* = 0.001). Additionally, Mostarac and Brajković (2022) suggest that the search for meaning explains approximately 20% of PTG, while Aflakseir et al. (2018) refers that MiL and social support together explain 34% of PTG.

In regard to the relationship between MM, only one study assessed its effects on PTG. Park et al. (2008) found a direct effect of MM on PTG, more specifically, they found this effect to be positive when cross-sectional (β = 0.44, *p* ≤ 0.05) but negative with a longitudinal design (β = −0.26, *p* ≤ 0.05). Nevertheless, MM at Time 1 and MM at Time 2 when combined explained 33% of the variance of PTG.

#### Religion and spirituality

MiL and PTG were assessed by some studies for their association with other variables, such as religion and spirituality. These studies pointed to a positive correlation between PTG, MiL, and MM and religion and spirituality.

George and Park (2013) suggest that MiL is positively correlated with religion and spirituality, adding that daily spiritual experiences have a positive effect on MiL (β = 0.28, *p* ≤ 0.05). Thuné-Boyle et al. (2011) found MM and PTG to be positively correlated to religion/spirituality. Kallay (2006) study on the other hand showed that one of the subscales of the Personal Meaning Profile, related to religious MM, and had a strong correlation with PTG (*r* = 0.70, *p* ≤ 0.01). Additionally, according to Moghadam et al. (2021) negative religious coping has a negative significant effect on PTG (β = −0.49, *p* ≤ 0.001).

#### Social support

Social support is more commonly associated with PTG. Indeed, four studies showed social support to be positively correlated with PTG (Thuné-Boyle et al., 2011; Aflakseir et al., 2018; Shand et al., 2018; Moghadam et al., 2021). Shand et al. (2018) added that certain domains of PTG, relating to others and appreciation of life, were associated with a higher perception of social support. Furthermore, Aflakseir et al. (2018) revealed that social support and MiL can explain 34% of the variance of PTG.

#### Anxiety and depression

Concerning MiL, higher levels of presence of meaning were not only associated with lower levels of anxiety and depression but predicted lower levels of depression after 1 year (β = −0.47, *p* ≤ 0.01) (Loeffler et al., 2018). PTG also showed to have a negative correlation with anxiety and depressive symptoms, indicating that lower levels of PTG were correlated with higher symptoms of depression and anxiety (Shand et al., 2018).

#### Life satisfaction

Three studies found a significant positive correlation between MiL and life satisfaction (George and Park, 2013; Loeffler et al., 2018; Mostarac and Brajković, 2022). PTG was also positively correlated with life satisfaction, explaining 46% with MiL as a mediator (Mostarac and Brajković, 2022). Through the presence of meaning, PTG shows an indirect effect on life satisfaction (β = 0.25, *p* ≤ 0.05). In fact, the presence of meaning in life explained more than 50% of the total effect of PTG on life satisfaction (Mostarac and Brajković, 2022).

### Quantitative synthesis: Meta-analysis

Considering that only two studies assess the concept of MM, and that the instruments used are not made to specifically measure the concept, only one meta-analysis was conducted between MiL and PTG. Table 2 shows the correlation coefficients extracted from the articles included in the systematic review, as well as the results from the quality assessment.

**Table 2:** Meaning in life and meaning-making correlations to posttraumatic growth.

Study (Authors, date)	Study design	MiL correlation coefficient (*r*)	MM correlation coefficient (*r*)	*N*	Quality assessment
Kallay (2006)	Cross-sectional	0.59	-	36	4
Park et al. (2008)	Cross-sectional	0.36	0.46	250	5
Thuné-Boyle et al., 2011	Cross-sectional	NR*	0.24	155	4
George and Park (2013)	Cross-sectional	0.32*	–	152	6
Loeffler et al. (2018)	Longitudinal	NR*	–	65	5
Shand et al. (2018)	Cross-sectional	NR*	–	108	4
Aflakseir et al. (2018)	Cross-sectional	0.33	–	196	5
Moghadam et al. (2021)	Cross-sectional	0.45	–	213	4
Mostarac and Brajković (2022)	Cross-sectional	0.44	–	149	6

NR, not reported; *Not included in meta-analysis.

From the nine studies considered, only five were included in the meta-analysis (*n* = 844). Three studies had lack of data which did not allow to determine the association between the variables (Thuné-Boyle et al., 2011; Loeffler et al., 2018; Shand et al., 2018). Two studies (Park et al., 2008; George and Park, 2013) shared the same sample. And so, to ensure the independence of the sample, the article with the smaller sample size was excluded (George and Park, 2013). Park et al. (2008) included results for two different times, only the first cross-sectional correlation was included. Kallay (2006) used two different instruments to measure PTG – Stress Related Growth Scale and Posttraumatic Growth Inventory. For that reason, the authors decided to include only the correlation with the PTGI, considering that it is the most used instrument for PTG in the included studies. Two articles had correlations between PTG and the presence and search for meaning (Moghadam et al., 2021; Mostarac and Brajković, 2022). Based on the construct of MiL defined in the inclusion criteria, only the data for the presence of meaning was considered.

#### Effect size

Table 3 shows the results obtained from the meta-analysis, including the main effect size for the correlation between MiL and PTG. MiL was significantly correlated with PTG, revealing a medium effect size (*r* = 0.43, 95% CI [0.36, 0.50], *p* ≤ 0.001).

**Table 3:** Mean effect sizes of meaning in life in posttraumatic growth.

	k	ES	95% CI	Z	Q	I^2^	Egger’s Test
Meaning in Life	5	0.43*	[0.36, 0.50]	11.9*	5.056	5.23%	1.615

k, number of studies; ES, effect size (mean weighted correlation coefficient); CI, confidence interval; Q, test of homogeneity; I^2^, proportion between studies variability; Egger’s test, Egger’s regression test of publication bias. *p < 0.001.

#### Heterogeneity

**Figure 2:**
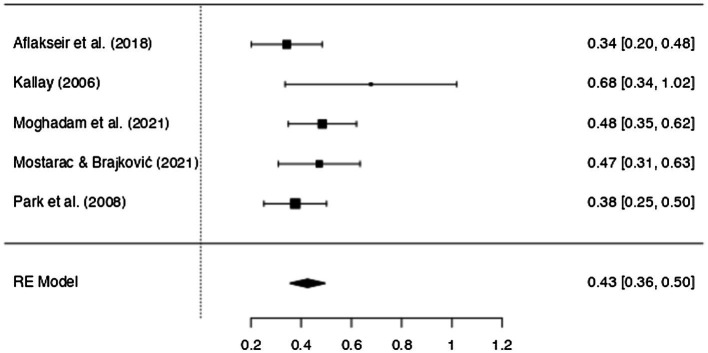
Forest plot for heterogeneity assessment (Source: JAMOVI).

The *Q* test suggested that there was no significant amount of heterogeneity (*Q* = 5.056, *df* = 4, *p* = 0.282), which suggests that the differences observed can be due to sampling error alone. The *I*^2^ was below 25% (*I*^2^ = 5.23), indicating a low level of heterogeneity and no substantial differences between studies. The forest plot represented in Figure 2 shows the correlation and confidence interval of each study, as well as their impact on the overall effect size represented in the last line. From the size of the black boxes, it is possible to say that the study of Kallay (2006) was the one with the smaller weight. Park et al. (2008) and Moghadam et al. (2021) studies had a bigger impact on the true effect size. The 95% confidence interval shows that the studies point to the same direction of the estimated mean effect.

#### Publication bias

The visual inspection of the funnel plot did not show an asymmetry, indicating the inexistence of bias due to the variability of the individual studies. Considering the small number of studies and the subjective nature of the funnel plot analysis, the Eggers Regression was used to confirm the absence of publication bias. The Egger’s test was not statistically significant (Egger’s test = 1.615, *p* = 0.106) confirming the examination of the funnel plot. The absence of publication bias strengthens the internal validity of the meta-analysis.

## Discussion

The aim of the present review was to analyze the empirical literature concerning meaning in life and PTG in adult cancer patients. The inclusion of a meta-analysis allowed to further assess the association between these two variables. To our knowledge, this is the first meta-analysis to evaluate the correlation between MiL and PTG, and to summarize the relationship found between these variables. A previous systematic review on psychological and clinical correlates of PTG in cancer patients suggested that meaning was linked PTG (Casellas-Grau et al., 2017). There was, however, no clear distinction and definition of the concept of MiL which resulted in the inclusion of articles that mostly considered PTG and meaning to be either synonyms or part of the same concept. Without a differentiation of these concepts, it is not possible to properly assess the influence and impact that they have on each other. When a study tries to examine the impact of one variable that is perceived as an integrant part of the other, then there is no surprise that they will both be correlated. It is the same as using two different instruments to study one and the same concept.

Meaning is a vital part of the human existence, while PTG is specific to the context of stressful life events. Seeing as they are distinct concepts with different implications, as was presented in the beginning of this article, this meta-analysis ensured that the inclusion criteria made a clear distinction of these concepts. In consequence, the studies were included only if they used separate and validated instruments to assess the concepts of MiL and PTG. It was this that allowed to extract and analyze solely the articles that perceived these variables as different concepts.

Research on psycho-oncology has studied the impact of MiL and PTG as independent variables. The results have pointed to a relationship between these variables and physical and mental health outcomes in cancer patients. However, there seems to be a growing interest in the relationship between these two variables. All the empirical studies included in this analysis revealed a significant positive correlation between MiL and PTG, resulting in an overall moderate effect size. In other words, a higher perception of MiL is associated with higher levels of PTG in cancer patients.

The ability to live meaningfully allows people to have a better perception of their life experiences, including their health. It is for this reason that meaning has been associated with better health indicators (Roepke et al., 2014) and increased well-being (Visser et al., 2010; Dezutter et al., 2013; García-Alandete, 2015; Krok, 2015). In cancer patients, MiL has been shown to have a positive impact on their illness perception (Krok and Telka, 2017; Krok et al., 2019), acceptance of cancer (Quinto et al., 2022), and in lowering the distress levels (Winger et al., 2016). The literature has showed that MiL is related to a number of factors that ultimately impact a cancer patient’ adjustment and experience. Some of the studies included in this systematic review have also shown a relationship between MiL and religion/spirituality, anxiety, depression and life satisfaction. Two of the included studies showed that not only is spirituality positively correlated (Thuné-Boyle et al., 2011) but also a predictor of higher levels of MiL (George and Park, 2013). Other studies have supported these findings. A study with cancer and heart failure patients found spirituality to have a positive impact on meaning (George and Park, 2017). While another study with advanced cancer patients showed that a higher MiL was associated with lower spiritual pain (Gravier et al., 2020). The relationship between these two variables appears, therefore, to go both ways. Spirituality, as a feeling of transcendence and connection, may facilitate a higher sense of MiL. On the other hand, experiencing MiL can also improve spiritual well-being in times of adversity. Regarding the relationship between MiL and anxiety and depression, Loeffler et al. (2018) found that MiL is a predictor of lower levels of anxiety and depression. This relationship has been evidenced in other studies with cancer patients (e.g., Vehling et al., 2011; Elekes, 2017; Dursun et al., 2022). There may be however a more complex connection between these three variables, specifically concerning the meaning of the illness itself. A longitudinal study with a focus on sources of meaning revealed that while most sources predicted lower levels of anxiety and depression, other meanings, such as ‘leaving a legacy’, predicted higher levels (Scheffold et al., 2014).

As the studies in this review show, MiL has a correlation to a variety of other variables besides PTG. And these variables can ultimately influence and impact the well-being and psychological adjustment to cancer. However, despite the numerous evidence regarding the relationship between MiL and mental health indicators, the literature would benefit from further examination of the mechanisms behind meaning. For instance, presence and search for MiL appear to have different and even inverse correlation with certain variables (Steger, 2018), such as well-being and cancer acceptance (Dezutter et al., 2013). This was evidenced in Moghadam et al. (2021) study, where search for meaning was negatively correlated with PTG, while the presence of MiL yielded a positive correlation. Some studies however do not show this inverse tendency, as in the study of Mostarac and Brajković (2022), where both presence and search for meaning where positively related to life satisfaction. The incoherence between studies can be due to the complexity of the interaction between presence and search for meaning. More comprehensive studies have suggested that a higher search for meaning can only lead to higher levels of well-being and life satisfaction when there is already a high presence of MiL. However, for a person with a low presence of MiL, the search for meaning can be a stressful experience that leads to a worse well-being, than experiencing a low presence and low search for meaning (Park et al., 2010; Dezutter et al., 2013; Krok and Telka, 2017). As it was stated before, meaning is a part of the human existence, it is how individuals are able to make sense of the world they live in. Without personal meaning, one will feel out of place or void, more commonly named a “meaningless existence.” In this case, when a person is faced with an adversity, as a cancer diagnosis, and there is a lack of foundation there will be an increased difficulty in coping and adjusting to the situation. This occurs as the news of cancer highlight the void of meaning, generating distress and consequently leading to symptoms of anxiety and depression. On the other hand, when there is a solid presence of meaning, the diagnosis of cancer can be distressful, but as one searches for meaning in the illness and adjusts this meaning to their overall MiL, the level of distress will subdue. Finding meaning in an adverse experience is the first step to not only adjust to it but possibly grow from it.

Posttraumatic growth is described as the ability to overcome and actually be able to experience a positive change from a highly stressful situation, such as cancer (Tedeschi and Calhoun, 1995; Tedeschi et al., 2018). The ability to grow from an experience can be considered the best outcome which is why in the last two decades the focus of research has shifted from posttraumatic stress to PTG. Numerous studies have explored the correlates and predictors of PTG in cancer patients, in an attempt to develop and guide psychosocial interventions that could lead these patients to a positive outcome. Several systematic reviews and meta-analysis have summarized and analyzed the literature on the subject (e.g., Casellas-Grau et al., 2017; Turner et al., 2018; Li et al., 2020; Yastıbaş and Karaman, 2021). In the present review the included studies found a positive correlation between PTG and spirituality (Kallay, 2006; Thuné-Boyle et al., 2011; Moghadam et al., 2021), social support (Thuné-Boyle et al., 2011; Aflakseir et al., 2018; Shand et al., 2018; Moghadam et al., 2021) and life satisfaction (Mostarac and Brajković, 2022). Other systematic reviews have also shown that higher levels of PTG are related to higher levels of positive outcomes, such as optimism, spirituality, positive affect and hope (Casellas-Grau et al., 2017; Turner et al., 2018; Yastıbaş and Karaman, 2021). Additionally, Casellas-Grau et al. (2017) systematic review revealed that most articles find a negative correlation between PTG and symptoms of depression and anxiety. This supports the findings of Shand et al. (2018), which suggest that PTG can ease depressive and anxious symptoms, so prevalent in the cancer population. Regarding the predictors of PTG, the most frequent in the literature is social support (Turner et al., 2018; Yastıbaş and Karaman, 2021), which explains why so many of the included studies in this review also revealed a positive correlation between these two variables.

As the scientific literature started to assess and see a relationship between MiL and PTG, the research begun to explore this relationship further in a variety of contexts, including cancer patients. The studies included in this review all pointed to a positive correlation between MiL and PTG in cancer patients. Three studies also showed a direct relationship between MiL and PTG (Aflakseir et al., 2018; Moghadam et al., 2021; Mostarac and Brajković, 2022). This suggests that MiL may play a role in facilitating and promoting PTG in cancer patients. Despite the fact that experiencing an adverse situation can be unsettling, and meaning can get called into question, it is the struggle to find meaning in cancer and to adjust to it that can promote personal change, in a variety of domains. Other studies have been able to find the same correlations across a variety of contexts and samples, such as survivors of natural disasters (e.g., Boullion et al., 2020; Weber et al., 2020; Dursun et al., 2022) or witnesses of extremely violent events (e.g., Aliche et al., 2019; Seol et al., 2021). In addition to cancer patients, the research on the relationship between MiL and PTG has also focused on similar experiences, like individuals with chronic illness (e.g., Zeligman et al., 2018; Wang et al., 2021).

There is undoubtedly a positive relationship between MiL and PTG. Higher levels of meaning, in people who have undergone a traumatic experience or an adverse situation, are associated with higher levels of PTG. There is still much to uncover regarding the relationship between these two variables, more specifically, how they relate and influence one another. In the context of an illness, it is also important to explore the possible differences caused by personal and illness characteristics (e.g., age, gender, illness stage and related symptoms).

Nevertheless, the research can only meaningfully impact the clinical practice if a consensus arises regarding the definition and distinction of these concepts. The scientific literature is particularly inconsistent in the conceptualization of MiL. Some use meaning as a synonym for spirituality, and some even see meaning as an equivalent of PTG. There is however a difference between experiencing MiL, finding meaning in an adverse situation or experiencing personal change caused by stressful situation. The model of meaning-making (Park and Folkman, 1997) seems to have brought confusion to the distinction between MiL and PTG. However, MiL, MM and PTG are all distinct concepts and should be seen as such. While MiL relates to the presence of meaning in one’s life, which includes a sense of purpose and a feeling of coherence and importance of life, MM is a process of search for meaning (Steger, 2018). Defined by Park and Folkman (1997), MM describes how a person can attempt to search for a meaning in a traumatic event that she can incorporate into her global MiL. On the other hand, PTG refers to the psychological processes that lead to an individual’s growth as a result from the struggle with a highly challenging circumstance (Tedeschi et al., 2018). In the process of PTG, it is during deliberate rumination that meaning can play a more active role by providing a meaning to the event that will facilitate its integration in the life narrative of the cancer patient. Additionally, having a strong sense of MiL previous to the diagnosis may also provide the necessary support and strength to only adjust to the cancer experience and find meaning in it, but also favor the development of personal growth.

Without a consensus or the awareness of these distinctions, we will keep seeing studies assess these concepts with different perspectives and measures, which will prevent us from truly grasping the impact and relationship between meaning and PTG in cancer patients or other populations. It is in fact the discrepancies between studies that represent the reason behind most of the limitations of this systematic review and meta-analysis.

### Limitations and future research

A meta-analysis can be performed as long as there are two studies (Valentine et al., 2010). Despite this, the inclusion of a small number of studies limits the strength and further analysis of the relationship between MiL, PTG and its correlates. Considering the heterogeneity of the cancer population it would be relevant to determine if there are significant differences when adding other factors to the analysis, such as age, gender, cancer type, stage or time since diagnosis. These individual differences and cancer characteristics were not taken into account due to the small number of studies. Regardless, these factors can influence either one of the variables, as it has been shown in the correlation found between time since diagnosis and PTG (Cordova et al., 2001; Danhauer et al., 2013; Cormio et al., 2017). It would be interesting to understand if and how individual, cultural and cancer differences have an influence on both MiL and PTG.

The divergences in the conceptualization of MiL also brought some limitations, especially when concerning the instruments used to assess meaning. Some studies applied the Functional Assessment of Chronic Illness Therapy – Spiritual Well-being scale (FACIT-SP), a scale that includes meaning but assesses predominantly spirituality. These studies had to be excluded seeing as they considered meaning to either be a synonym or a component of spirituality (e.g., Park and Cho, 2017; Bi et al., 2021). The included studies applied different instruments to assess MiL, which can be a threat to the validity of the results obtained by the meta-analysis. Three out of five studies used the Meaning in Life Questionnaire (MILQ) to assess MiL. Additionally, these are also the most recent articles, which points to a tendency for the use of this instrument. Therefore, in the future the use of the MILQ would benefit the research of MiL.

Meaning-making is a complex process that involves a variety of components. But unlike PTG, there is no validated instrument that measures the entirety of the process or its outcomes (meanings made). For that reason, there are fewer studies that assess this variable. The two studies that measured MM and PTG were not considered for meta-analysis, due to the lack of a valid instrument for the concept. Taking into consideration that MM is a process, rather than an outcome, a longitudinal approach would be more fitting for future research. Additionally, it should be considered the possibility of the development of an instrument to measure the cognitive processes that underlie MM.

### Clinical implications

Meaning is undoubtedly related to PTG in cancer patients. Both MiL and PTG have shown a positive impact in several psychological outcomes, such as anxiety, depression and life satisfaction. Cancer patients have a higher risk of developing psychological disorders, specifically those related with anxiety and depression (Caruso et al., 2017). As our findings have showed MiL can have a positive or negative role in the adjustment to the illness, facilitating or not PTG. All the included studies indicated that higher levels of MiL are associated to also higher levels of PTG. On the other hand, literature has shown that the absence of meaning can not only be distressful but lead to worse health outcomes. The ability to detect patients struggling with meaning can be the key to assist these patients adjustment to cancer, by suggesting psychology support or specialized meaning-centered interventions. The existing distress protocols do not include existential problems, such as trouble in finding the meaning of cancer. Seeing as a lack of meaning can jeopardize the emotional well-being, it would be relevant to give these patients the opportunity to express such personal concerns when there are higher levels of distress. Regarding psychotherapeutic interventions centered on meaning for cancer patients, these would benefit from considering the correlates and predictors of PTG, in order to better assist them to achieve personal growth. Meaning-centered interventions are mostly applied to advance or terminal cancer patients (Breitbart, 2002; Mok et al., 2012; Guerrero-Torrelles et al., 2017; Kang et al., 2019). The struggles with meaning, however, can emerge in all cancer patients, even those that are not in advanced stage. Meaning-centered interventions in these patients could facilitate a better adjustment to the illness, as well as to life after cancer. For that reason, further studies on the impact of meaning throughout the different stages of cancer, that also include an analysis to the illness and individual differences (e.g., type of cancer, age, gender), may benefit the application of these interventions in all the cancer patients that show an interest and need for support in the future. Additionally, considering the role of meaning in a cancer patient experience and their potential growth, it would be relevant to develop an intervention that, in addition to its focus on meaning, incorporated the processes of PTG in order to facilitate personal growth. This intervention could explore an individual’s pre-existing MiL and make use of their personal resources to assist and guide the search for meaning in cancer, while taking into consideration the sociodemographic and psychosocial characteristics and cognitive processes described by the model of PTG. This way, the more existential concerns of the patient could be addressed while providing room for the development of growth.

## Data availability statement

The original contributions presented in the study are included in the article/Supplementary material, further inquiries can be directed to the corresponding author.

## Author contributions

MA conceived and designed the study, collected and analyzed the data, and drafted the manuscript. CR analyzed the data, supervised the overall process, and revised the manuscript. LM and MB-P consulted and assisted in the meta-analysis procedure. IL supervised the study. All authors contributed to the review and writing of the final manuscript.

## Funding

This work is funded with national funds from FCT – Fundação para a Ciência e Tecnologia, I.P., in the context of the project UID/04810/2020.

## Conflict of interest

The authors declare that the research was conducted in the absence of any commercial or financial relationships that could be construed as a potential conflict of interest.

## Publisher’s note

All claims expressed in this article are solely those of the authors and do not necessarily represent those of their affiliated organizations, or those of the publisher, the editors and the reviewers. Any product that may be evaluated in this article, or claim that may be made by its manufacturer, is not guaranteed or endorsed by the publisher.
