# Supplementary material for: Meaning in life, meaning-making and posttraumatic growth in cancer patients: Systematic review and meta-analysis
Source: Front Psychol. 2022 Dec 9;13:995981. doi: 10.3389/fpsyg.2022.995981 (PMC9784472; doi:10.3389/fpsyg.2022.995981)
Supplement: Supplementary file 1 [file Table_1.docx]

**Supplementary Table.** Search strategy applied in databases

| **Databases** | **Search by title and abstract** |
| --- | --- |
| Academic Search Complete, Complementary Index, MEDLINE, APA PsycINFO, ScienceDirect, Psychology and Behavioral Sciences Collection, Supplemental Index, Directory of Open Access Journals, APA PsycArticles, ERIC, Business Source Complete, Criminal Justice Abstracts, Library Information Science & Technology Abst, RCAAP, PubMed, Bon and Web of Science | Cancer OR Oncological Disease OR Neoplasm OR Tumour OR Tumor AND Posttraumatic Growth OR Post-Traumatic Growth OR Benefit Finding OR Positive Life Changes OR Stress-related Growth OR Perceived Benefits OR Existential Growth AND Meaning* OR Existential Meaning OR Purpose OR Meaning-making OR Meaning Making OR Search* for meaning |
| SCOPUS | Meaning* AND Posttraumatic Growth AND Cancer |
|  | Meaning* AND Benefit Finding AND Cancer |
|  | Meaning* AND Stress Related Growth AND Cancer |
|  | Purpose AND Posttraumatic Growth AND Cancer |
| Scielo | Posttraumatic Growth AND Cancer |
|  | Meaning AND Benefit AND Cancer |
